# General practitioners’ evaluations of optimal timing to initiate advance care planning for patients with cancer, organ failure, or multimorbidity: A health records survey study

**DOI:** 10.1177/02692163211068692

**Published:** 2021-12-30

**Authors:** Willemijn Tros, Jenny T van der Steen, Janine Liefers, Reinier Akkermans, Henk Schers, Mattijs E Numans, Petra G van Peet, A. Stef Groenewoud

**Affiliations:** 1 Department of Public Health and Primary Care, Leiden University Medical Center (LUMC), Leiden, The Netherlands; 2 Department of Primary and Community Care, Radboud University Medical Center, Nijmegen, The Netherlands; 3 Radboud Institute for Health Sciences, Scientific Center for Quality of Healthcare, Radboud University Medical Center, Nijmegen, The Netherlands

**Keywords:** Advance care planning, cancer, organ failure, multimorbidity, general practice, electronic health record, surveys and questionnaire

## Abstract

**Background:**

Appropriate timing to initiate advance care planning is difficult, especially for individuals with non-malignant disease in community settings.

**Aim:**

To identify the optimal moment for, and reasons to initiate advance care planning in different illness trajectories.

**Design and methods:**

A health records survey study; health records were presented to 83 GPs with request to indicate and substantiate what they considered optimal advance care planning timing within the 2 years before death. We used quantitative and qualitative analyses.

**Setting and patients:**

We selected and anonymized 90 health records of patients who died with cancer, organ failure or multimorbidity, from a regional primary care registration database in the Netherlands.

**Results:**

The median optimal advance care planning timing according to the GPs was 228 days before death (interquartile range 392). This moment was closer to death for cancer (87.5 days before death, IQR 302) than for organ failure (266 days before death, IQR 401) and multimorbidity (290 days before death, IQR 389) (*p* < 0.001). The most frequently mentioned reason for cancer was “receiving a diagnosis” (21.5%), for organ failure it was “after a period of illness” (14.7%), and for multimorbidity it was “age” and “patients” expressed wishes or reflections’ (both 12.0%).

**Conclusion:**

The optimal advance care planning timing and reasons to initiate advance care planning indicated by GPs differ between patients with cancer and other illnesses, and they also differ between GPs. This suggests that “the” optimal timing for ACP should be seen as a “window of opportunity” for the different disease trajectories.

**What is already known about the topic?**

- GPs find it difficult to determine the right time to initiate advance care planning, especially in patients with non-malignant diseases.
- Appropriate timing of advance care planning is important, as initiating it too early could lead to plans not reflecting patient wishes accurately, and initiating it too late could result in rushed decisions about end-of-life care.

**What this paper adds?**

- Perceptions of the optimal time to initiate advance care planning differ among GPs and also for patients with cancer and those with organ failure or multimorbidity.
- The optimal timing to initiate advance care planning could be seen as a “window of opportunity.”
- We identified GPs’ reasons for and factors contributing to the decision to initiate advance care planning, such as “patients” expressed wishes or reflections’, “receiving a diagnosis,” “after a period of illness,” and “age.”

**Implication for practice, theory, or policy**

- Reasons GPs provide to initiate advance care planning could flag the start of a “window of opportunity.”
- GPs could consider initiating advance care planning when patients show readiness, at a moment when there is time to discuss it and the patient is in a relatively good condition.
- It is important to realize “not one size fits all,” the timing of advance care planning must be tailormade.

## Introduction

Advance care planning is a process that enables patients to specify and discuss their goals and preferences for future treatment and care with their healthcare providers and family. The process also encourages the timely review of these preferences.^1,2^ Research has shown that advance care planning may improve the quality of end-of-life care^3^ and improves concordance between patients’ preferred and received end-of-life care.^4–6^ Advance care planning increased hospice care and reduced the number of hospitalizations.^3^ However, these findings should be interpreted carefully due to the diversity of populations and methodological quality constraints.^3,7^ All in all, benefits of and doubts about advance care planning are still subject of debate^8–10^ which may have prevented its broad implementation in general practice.^11,12^ Meeussen et al.^11^ found that in the Netherlands and Belgium advance care planning was used in 34% of patients with non-sudden death. Another study found that General Practitioners (GPs) discussed all topics around end of life more frequently with cancer patients than with patients with organ failure or old age.^12^

This may relate to the difficulty of deciding when to initiate advance care planning in case of organ failure or old age.^13–15^ The life-limiting nature of non-malignant disease such as heart failure is not always apparent, which make it difficult to define key moments to initiate advance care planning.^13^ Furthermore, GPs are concerned about inducing anxiety in patients with non-malignant disease by making them aware of their diagnosis and prognosis.^13,15^

Nevertheless, optimal timing of advance care planning is important, as patients’ preferences vary over time and choices about life-sustaining treatments depend on context. Initiating advance care planning too early as a one-time event risks that plans do not accurately reflect patients’ wishes over time.^16^ Also, for some patients communication about the end of life shortly after diagnosis is too soon and distressing.^17^ On the other hand, starting advance care planning too close to the end of life could lead to rushed decisions lacking focus on underlying values, preferences and goals.^1,16^

Indicators and tools have been developed to support identification of patients in need of palliative care.^18–20^ However, as yet there are no practical tools or clinical indicators to identify patients in need of advance care planning, despite research showing that GPs would appreciate such guidance.^16^

### Objectives

We aimed to determine what GPs, based on the assessment of routine electronic health records of patients with cancer, organ failure or multimorbidity, consider the optimal advance care planning timing and important clinical indicators to initiate it. Additionally, we investigated whether these differed between the three different groups and between GPs, and whether an association existed between patient and GP characteristics and optimal advance care planning timing.

## Methods

### Study design and setting

We conducted a health records review study. We presented anonymized routine electronic patient health records, collected from GPs, of patients who died with cancer, organ failure or multimorbidity to randomly sampled other GPs, asking the latter to indicate what they considered to be the optimal time to initiate advance care planning.

### Data source

We selected pseudonymized patient health records from the database of FaMe-net, a primary care registration network in the region of Nijmegen, in the eastern part of the Netherlands. This network routinely collects patient data from seven different general practices and provides access to these data for research purposes. We selected 1235 health records from patients who had died between 2003 and 2016 that included documentation of the last two years before death. The patient health records contain personal characteristics, reports from the GPs, correspondence to and from other healthcare providers (specialists, out-of-hours primary care, and paramedic services), laboratory values and medication prescriptions.

First, we excluded all records of patients under the age of 18 and patients diagnosed with dementia, as advance care planning in patients with dementia needs its own approach.^21,22^ Additionally, we excluded records with missing or incomplete data.

Second, we randomly sampled 150 patient health records from the database, distributed equally across the seven general practices. Within this selection, we assigned records with diagnoses verified from their recorded medical history to three different groups, based on the different illness trajectories described by Murray et al.^23^ and Lynn and Adamson^24^: (i) patients who died with cancer, whose decline is generally evident and progressive. (ii) patients who died with organ failure (COPD, heart failure, kidney failure, liver failure, and chronic-progressive neurological illness such as Parkinson or ALS), whose decline is characterized by long-term limitations with intermittent worsening of symptoms and some recovery, with often a rather sudden death. (iii) older patients who died with multiple (>2) chronic diseases, other than cancer and organ failure (i.e. multimorbidity), whose decline is generally prolonged and gradual. We excluded patients with a sudden death (unpredictable and acute illness or trauma).

Third, we randomly added patients to the three different groups until there were 30 in each group, based on the central limit theorem, to create a normal distribution of means as a requirement for valid application of parametric statistics.^25^

Finally, we manually anonymized these 90 patient health records by removing all information that referred to individuals, and we left out information reported by GPs regarding practical application of advance care planning. We pilot tested the presentation of the patient health records in the online environment with four GPs.

### Recruitment of participants

We recruited 90 GPs from various networks. They were asked to examine three patient health records (one from each of the three groups). Each patient health record was presented to three GPs, which allowed for evaluation of the agreement between GPs on optimal advance care planning timing.

We recruited GPs from networks with and without a particular interest in end-of-life care from various geographical areas in the Netherlands (reach of approximately 970 GPs). We recruited GPs through primary care networks that focus on education and research collaborate. We also invited a group of former participants of an academic course “Ethics in General Practice” to participate in the study. Further, PalHAG, an association of GPs specialized in palliative care, published an invitation to active members on the PalHAG website. Last, we asked GPs to disseminate the invitation to participate in our study in their own professional networks (snowball sampling).

### Data collection

GPs who expressed an interest by email received an extensive information letter about our study and user instructions and access to the online environment through a unique link. If they did not respond or the assessment was incomplete, we sent a reminder 10 days, and if necessary 17 days after enlisting. Data were collected between 31 October 2020 and 10 January 2021.

Our online questionnaire comprised: (i) informed consent form; (ii) short questionnaire for demographic information (Supplemental File 1); (iii) three anonymized patient health records.

The patient health records consisted of all documentation of health care provided during the last 2 years before death, depicted in a “scrollable” timeline (see Supplemental File 2 for an example). For each patient health record GPs were asked to identify the optimal advance care planning timing, and to briefly substantiate this choice in free text. We furthermore asked GPs to indicate other information in the health record that they viewed as contributing factors in their determination of the optimal advance care planning timing, and to motivate this as well.

### Data analysis

Patient and respondent characteristics were assessed using descriptive statistics.

We determined mean and standard deviation (SD) or median and interquartile range (IQR) for continuous characteristics and number and percentages for categorical characteristics. We present optimal advance care planning timing as the median of days and IQR between the assigned optimal moment and patients’ death from all reviewed health records. Agreement was reached when the three GPs who reviewed the same health record all assigned the optimal advance care planning timing within a maximum range of 30 days from each other, and partial agreement was reached when 2 of the 3 GPs did. Differences between the three groups (cancer, organ failure, and multimorbidity) were tested using ANOVA (in case of normal distribution) or the Kruskall Wallis test (in case of skewed distribution). Differences in advance care planning timing between GPs with specific characteristics (gender and expertise in palliative care, care for older people, and chronic disease) and between male and female patients were tested using *t*-tests (in case of normal distribution) or the Mann-Whitney *U* test (in case of skewed distribution). A *p*-value of <0.05, based on two-sided tests was considered statistically significant for all analyses.

To determine which clinical indicators GPs find important for initiating advance care planning in the three groups, we analyzed the free text data with qualitative methods. We used inductive content analysis to derive categories and themes from the free texts^26–28^ The content analyses included a summative approach to the data. Two researchers (WT and SG) independently coded a first set of free texts, compared codes, and in case of divergences they consulted a third researcher (PP). We discussed the codes with the research team and modified or merged codes and formulated categories and themes. Afterward, WT applied the final codes to all data and SG randomly checked part of the coding. We performed quantitative analyses in SPSS version 25 (IBM, 2017) and qualitative analysis in Microsoft Excel 2016.

### Ethical aspects

The research ethics committee of the Radboud university medical center (CMO Radboudumc) approved the research protocol (number: 2018-4589). The Radboudumc Technology Center Health Data supports FaMe-Net in extraction and secure storage of routine data from the affiliated practices. It adheres to the regulations of Dutch and European laws and has ethical approval from the CMO Radboudumc for this procedure (number 2020-6871). Under Dutch and European privacy laws, it is not necessary to obtain informed consent for retrospective studies with anonymized patient data.

## Results

We recruited 94 GPs, of whom four withdrew, four did not respond, two responded after closure of data collection and one GP assessed the records incorrectly. This resulted in inclusion of 83 GPs who completed at least one valid evaluation of the three health record. In total, 247 health records evaluations were performed; 70 records were assessed by three GPs, 17 records by two GPs and three records by only one GP (on average, each record was assessed 2.74 times). The characteristics of the patients whose health records were used for analysis and of the participating GPs are shown in Tables 1 and 2.

**Table 1.** Characteristics of patients whose health records were used for analysis, *N* = 90.

	Total (*n* = 90)	Cancer (*n* = 30)	Organ failure (*n* = 30)	Multimorbidity (*n* = 30)
Sex: % (*n*)				
Female	51 (46)	53 (16)	47 (14)	53 (16)
Age in years at time of death: mean (SD)				
	81 (12)	71 (14)	83 (8)	87 (7)

**Table 2.** Characteristics of participating GPs, *N* = 83.

	Total
Sex: % (*n*)	
Female	74 (61)
Work experience as a GP in years: mean (SD)	
	15 (10)
Additional expertise (more possible, *n* = 96): % (*n*)	
Care for older people	17 (13)
Palliative care	24 (20)
Chronic diseases	7 (6)
Other additional expertise	24 (20)
No additional expertise	44 (37)
Type of practice: % (*n*)	
Solo practice	22 (18)
Duo practice	31 (26)
Group practice	47 (39)
Type of employment: % (*n*)	
Salaried service	13 (11)
Independent	59 (49)
Other	28 (23)
Practice location: % (*n*)	
Rural	11 (9)
Semirural	36 (30)
Urban	53 (44)

SD: standard deviation.

### Optimal advance care planning timing

**Figure 1.**
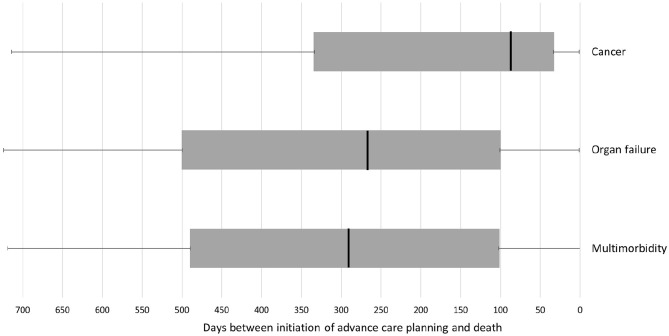
Optimal advance care planning timing as determined by the participating GPs. *N* = 245, excluding two records for which no optimal advance care planning timing was determined. Cancer, median (IQR): 87.5 (302); Organ failure, median (IQR): 266 (401); Multimorbidity, median (IQR): 290 (389). *p* value <0.001 (Kruskal-Wallis test).

Figure 1 displays the optimal advance care planning timing as identified by the GPs. The median optimal advance care planning timing assigned was 228 days (IQR 392) before death. The assigned moment was closer to death for the patients with cancer (87.5 days before death) than for the patients with organ failure (266 days before death) and multimorbidity (290 days before death) (*p* < 0.001).

**Figure 2.**
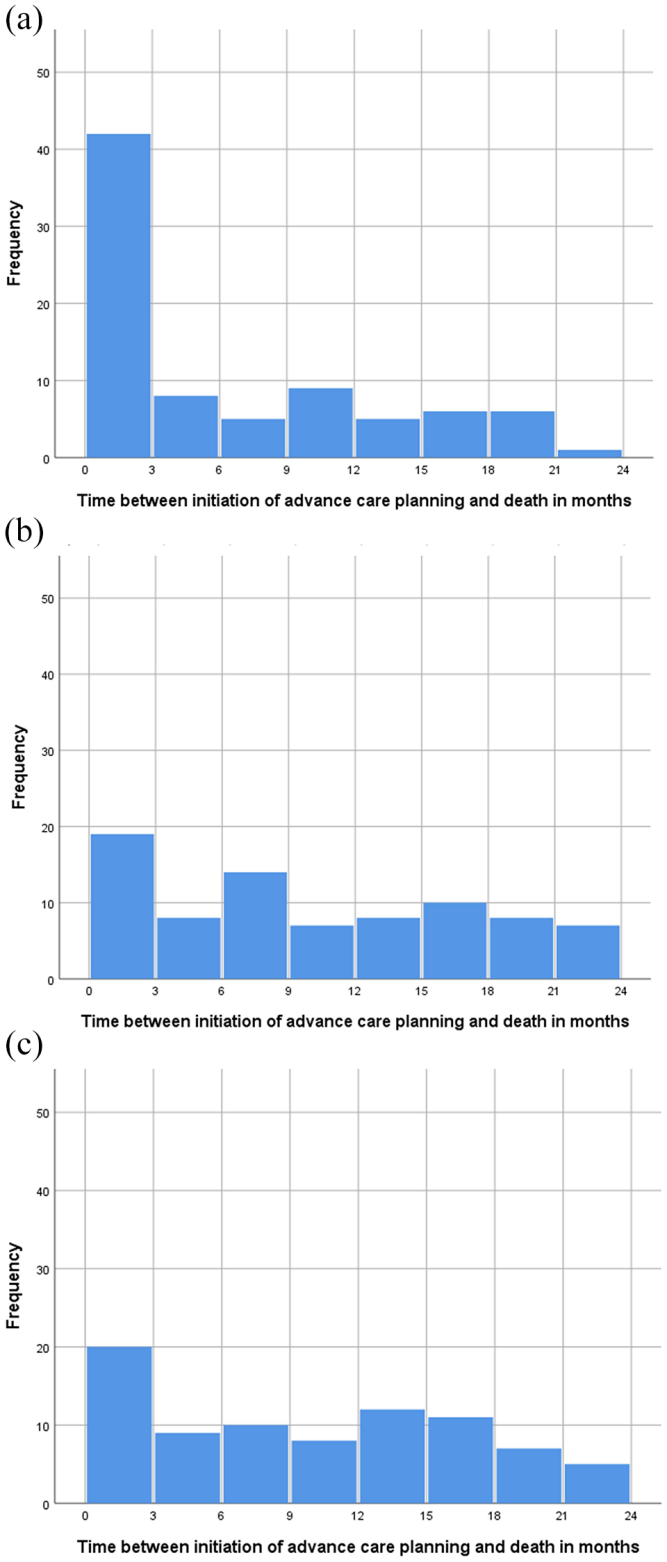
Time between optimal moment to initiate advance care planning and death in months: (a) cancer, n = 82, (b) organ failure, n = 81, and (c) multimorbidity, n = 82.

Figure 2 shows the optimal advance care planning timing in months per patient group as indicated by GPs. In 42% of the reviewed health records of patients with cancer (*n* = 82) the optimal advance care planning timing was assigned within 3 months before death, compared to 19% and 20% in patients with organ failure (*n* = 81), or multimorbidity (*n* = 82) respectively.

### Reasons and contributing factors to determine optimal time to initiate advance care planning

As shown in Table 3, the GPs indicated a total of 444 reasons for initiation of advance care planning. We clustered these reasons into seven themes and 27 categories. In general, the most frequently mentioned reasons were “expressions of patients” reflections or wishes’ (9.7%), followed by an “appropriate setting (e.g. period of relative wellness, a setting with adequate time or presence of a family member)” and “receiving a diagnosis” (both 8.8%). For patients with cancer, the most frequent reasons were “receiving a diagnosis” (21.5%), followed by “no curative treatment options available” (16.9%), having “a poor prognosis” (9.2%) and “expressions of patients” reflections or wishes’ (9.2%). For patients with organ failure these were “after period of illness” (14.7%), an “appropriate setting” and “exacerbation of organ failure” (8.8%) and for patients with multimorbidity, “age” (12.0%) and “expressions of patients” reflections or wishes’ (12.0%), followed by presence of “acute symptoms” (8.3%) and an “appropriate setting” (8.3%).

**Table 3.** Reasons for and factors contributing to optimal timing to initiate advance care planning.

	Total		Cancer		Organ failure		Multimorbidity	
	Reasons for initiating ACP	Factors contributing to initiating ACP	Reasons for initiating ACP	Factors contributing to initiating ACP	Reasons for initiating ACP	Factors contributing to initiating ACP	Reasons for initiating ACP	Factors contributing to initiating ACP
	*N = 444*	*N = 504*	*N = 130*	*N = 227*	*N = 170*	*N = 169*	*N = 144*	*N = 108*
In timeline of the disease: % (*n*)	34.9 (155)	29.2 (147)	56.9 (74)	41.0 (93)	30.6 (52)	22.5 (38)	20.1 (29)	16.7(18)
Diagnosis	8.8 (39)	6.3 (32)	21.5 (28)	11.9 (27)	3.5 (6)	2.4 (4)	3.5 (5)	0.9 (1)
After period of sickness	8.3 (37)	6.2 (31)	3.8 (5)	4.0 (9)	14.7 (25)	9.5 (16)	4.9 (7)	5.6 (6)
No curative treatment options	5.6 (25)	2.8 (14)	16.9 (22)	5.7 (13)	0.6 (1)	0.6 (1)	1.4 (2)	0 (0)
Poor prognosis	5.6 (25)	3.2 (16)	9.2 (12)	4.0 (9)	4.1 (7)	3.0 (5)	4.2 (6)	1.9 (2)
* S*uspicion of severe illness	3.2 (14)	5.0 (25)	2.3 (3)	6.2 (14)	3.5 (6)	1.8 (3)	3.5 (5)	7.4 (8)
Start of treatment or diagnostics	2.0 (9)	5.6 (28)	1.5 (2)	9.3 (21)	3.8 (5)	4.7 (8)	1.4 (2)	0.9 (1)
Unpredictable course of illness	0.7 (3)	0.2 (1)	1.5 (2)	0 (0)	0.6 (1)	0.6 (1)	0 (0)	0 (0)
The “surprise question” can be answered with “yes” or “maybe”^a^	0.7 (3)	0 (0)	0 (0)	0 (0)	0.6 (1)	0 (0)	1.4 (2)	0 (0)
Symptoms or behavior indicating deterioration: % (*n*)	27.5 (122)	32.9 (166)	13.1 (17)	22.5 (51)	30.0 (51)	45.6 (77)	37.5 (54)	35.2 (38)
Functional deterioration	5.2 (23)	3.8 (19)	0.8 (1)	2.6 (6)	6.5 (11)	3.6 (6)	7.6 (11)	6.5 (7)
Acute symptoms	4.7 (21)	6.2 (31)	3.1 (4)	3.5 (8)	3.8 (5)	8.9 (15)	8.3 (12)	7.4 (8)
Exacerbation of organ failure	4.1 (18)	3.6 (18)	0.8 (1)	1.3 (3)	8.8 (15)	8.9 (15)	1.4 (2)	0 (0)
General deterioration	4.1 (18)	3.2 (16)	0.8 (1)	2.6 (6)	4.7 (8)	3.0 (5)	6.3 (9)	4.6 (5)
“Red flag” symptoms	3.6 (16)	5.0 (25)	3.8 (5)	4.4 (10)	1.2 (2)	5.3 (9)	6.3 (9)	5.6 (6)
Deterioration of chronic disease	3.2 (14)	8.0 (40)	1.5 (2)	4.8 (11)	3.5 (6)	12.4 (21)	4.2 (6)	7.4 (8)
Cognitive deterioration	2.5 (11)	1.8 (9)	2.3 (3)	1.8 (4)	2.4 (4)	1.8 (3)	2.8 (4)	1.9 (2)
Change in consulting behavior	0.2 (1)	1.6 (8)	0 (0)	1.3 (3)	0 (0)	1.8 (3)	0.7 (1)	1.9 (2)
Mental/spiritual health aspects: % (*n*)	12.4 (55)	18.3 (92)	13.1 (17)	24.2 (55)	11.2 (19)	11.2 (19)	13.2 (19)	16.7 (18)
Expression of patients’ reflections or wishes	9.7 (43)	11.7 (59)	9.2 (12)	13.7 (31)	8.2 (14)	8.3 (14)	12.0 (17)	13.0 (14)
Expression of patients’ or family members’ emotions	2.0 (9)	6.2 (31)	3.1 (4)	10.1 (23)	1.8 (3)	2.4 (4)	1.4 (2)	3.7 (4)
Nature of patient	0.7 (3)	0.4 (2)	0.8 (1)	0.4 (1)	1.2 (2)	0.6 (1)	0 (0)	0 (0)
Patient characteristics: % (*n*)	10.6 (47)	11.9 (60)	6.2 (8)	7.9 (18)	11.2 (19)	10.1 (17)	13.9 (20)	23.1 (25)
Age	7.2 (32)	3.8 (19)	2.3 (3)	0.9 (2)	7.1 (12)	3.0 (5)	12.0 (17)	11.1 (12)
Extensive medical history	2.3 (10)	3.6 (18)	1.5 (2)	2.6 (6)	4.1 (7)	4.1 (7)	0.7 (1)	4.6 (5)
Medication use	1.1 (5)	4.6 (23)	2.3 (3)	4.4 (10)	0 (0)	3.0 (5)	1.4 (2)	7.4 (8)
Appropriate setting: % (*n*)	8.8 (39)	2.4 (12)	7.7 (10)	1.8 (4)	10.0 (17)	3.0 (5)	8.3 (12)	2.8 (3)
Social context: % (*n*)	2.9 (13)	1.8 (9)	0 (0)	1.8 (4)	3.5 (6)	2.4 (4)	4.9 (7)	2.8 (3)
Death or disease of partner or family member	1.6 (7)	1.4 (7)	0 (0)	0.9 (2)	1.2 (2)	1.2 (2)	3.5 (5)	2.8 (3)
Social vulnerability	0.7 (3)	0 (0)	0 (0)	0 (0)	1.2 (2)	0 (0)	0.7 (1)	0 (0)
Change of main healthcare professional	0.7 (3)	0.4 (2)	0 (0)	0 (0)	1.2 (2)	1.2 (2)	0.7 (1)	0 (0)
Signal to initiate ACP from other healthcare professional or family member: % (*n*)	2.9 (13)	3.2 (16)	3.1 (4)	1.8 (4)	3.5 (6)	5.3 (9)	2.1 (3)	2.8 (3)

ACP: advance care planning.

a The surprise question: “Would I be surprised if this patient died within 12 months?.” This can be used to identify patients at high one-year mortality risk.^29^

In addition, the GPs indicated a total of 504 factors that contributed to their conviction that advance care planning should be initiated. We allocated these factors to the same themes and categories. Of these factors, 11.7% were “expressions of patients” reflections or wishes’, followed by “deterioration of chronic disease” (8.0%) and “receiving a diagnosis” (6.3%).

### Association between GP- and patient characteristics and optimal advance care planning timing

The median optimal advance care planning timing in days before death was not associated with sex of the GP or patient or with additional expertise of the GP.

### The extent of agreement between GPs on optimal advance care planning timing

Table 4 demonstrates that there was agreement between all GPs on optimal advance care planning timing in 21% of the health records reviewed by three GPs. There was partial agreement on 47% of the health records reviewed by three GPs and no agreement on 32%. On health records regarding patients with cancer, organ failure and multimorbidity there was agreement between three GPs in 32%, 4%, and 26% of cases respectively.

**Table 4.** Percentage of agreement between GPs on the optimal advance care planning timing in health records reviewed by three GPs.

	Total (%)	Cancer (%)	Organ failure (%)	Multimorbidity (%)
Agreement	21^a^	32^b^	4^c^	26^d^
Partial agreement	47^e^	41^f^	57^g^	44^h^
No agreement	32^i^	27^j^	39^k^	30^l^

GP: general practitioner.

a 14/68.

b 7/22.

c 1/23.

d 6/23.

e 32/68.

f 9/22.

g 13/23.

h 10/23.

i 22/68.

j 6/22.

k 9/23.

l 7/23.

Fisher’s exact test: *p* = 0.178.

## Discussion

The GPs in our study determined the median optimal advance care planning timing to be 228 days before death (IQR 392). We found considerable differences regarding optimal advance care planning timing as determined by GPs as well as low agreement between GPs. Comparing the optimal advance care planning timing between the three groups showed a median optimal advance care planning initiation time closer to death for patients with cancer than for patients with organ failure and multimorbidity.

We identified several reasons GPs had for selecting optimal times to initiate advance care planning, including “expressions of patients” reflections or wishes,’ “appropriate setting” and “receiving a diagnosis.” For patients with cancer, “receiving a diagnosis” was the most important reason, for patients with organ failure it was “after a period of illness,” and for patients with multimorbidity “age” and “expressions of patients” reflections or wishes’ were the most important reasons.

### Comparison with existing literature

Our findings are in line with previous research that identified distinct approaches, perspectives and rationales of discussing advance directives by GPs, concluding that there is no “right” moment to initiate advance care planning.^30^ Furthermore, in another study, GPs emphasized the importance of tailoring advance care planning to each individual patient, which probably contributed to the widely different timing of advance care planning.^13^

The considerable difference in median advance care planning timing between the three groups might be due to the generally shorter duration of the disease and period of evident decline in cancer, compared to the longer progressive decline in organ failure and multimorbidity.^23^

The reasons we found for initiating advance care planning resemble the few previously identified indicators such as diagnosis and exhausted curative treatment options in cancer patients.^15,31^ A substantial number of GPs in our study suggested initiating advance care planning in “an appropriate setting,” which included a period of relative wellness. This approach is also suggested by healthy older patients.^32^

Studies and tools for the identification of patients in need of palliative care,^18,19,33^ although not the same as advance care planning, include clinical indicators similar to the indicators we found, such as (frequent) hospital admissions, progressive decline in functioning, and correspondence from medical specialists that cure is not longer possible. Our results add to previous research in that they suggest it is most important to GPs to initiate advance care planning after patients themselves express reflections and wishes, that is, when patients show a certain readiness.

### Strengths and limitations

To our best knowledge, this is the first health records review study to investigate the optimal timing and reasons for initiating advance care planning according to GPs in different illness trajectories. Another strength of our study is that we studied both optimal advance care planning timing, as well as reasons for inititiating advance care planning according to GPs. Furthermore, the retrospective design allowed GPs to evaluate time up to the end of life. However, this follow back perspective differs from practice when the end of life is not yet known. Other limitations include that only the last two years before death could be reviewed by the GPs. However, the optimal advance care planning timing was usually defined well within the limit of two years before death. Further, in those two years, the course of illness varied between individual patients. However, examining these differences was outside the scope of our study. Last, we included a high percentage of GPs with additional expertise in care for older people or palliative care, which implies we may even underestimate divergence in optimal advance care planning timing.

### Implication for practice

Our results indicate that GPs may perceive the optimal timing for advance care planning as a “window of opportunity,” instead of one clearly defined point in time that can be missed. GPs could consider initiating advance care planning when patients show readiness, at a moment when there is time to discuss it and the patient is in a period of relatively good condition, particularly early and pro-actively. Furthermore, our study shows the importance of distinguishing between the different disease trajectories when deciding upon advance care planning timing. In cancer patients, initiating advance care planning should be considered at the time of diagnosis; in patients with organ failure after a period of illness/exacerbation (such as a hospital admission); in patients with multimorbidity, higher age and symptoms indicating functional and general deterioration (such as decreasing mobility, increasing dependence, increasing fatigue and losing appetite) are more important and could be a reason to initiate advance care planning. As the name advance care planning already suggests, it involves a *proactive* approach. Furthermore, it is important for professionals and policymakers to realize that “not one size fits all,” and the timing of advance care planning must be tailormade.

### Future research

Our study could be a start for the development of practical tools to support GPs in deciding when to initiate advance care planning. However if our results would be used, for example, for the future development of an artficial intellegence algortihm, such an algorithm must be validated in general practice. In addition, further research will have to evaluate whether using such applications results in more and timely implementiation of advance care planning, eventually improving end-of-life care. Initiating advance care planning from a certain age for patients without a particular life-limiting disease could be part of a public health approach. This requires further study on satisfaction and effectiveness.

## Supplemental Material

sj-pdf-1-pmj-10.1177_02692163211068692 – Supplemental material for General practitioners’ evaluations of optimal timing to initiate advance care planning for patients with cancer, organ failure, or multimorbidity: A health records survey study Click here for additional data file. Supplemental material, sj-pdf-1-pmj-10.1177_02692163211068692 for General practitioners’ evaluations of optimal timing to initiate advance care planning for patients with cancer, organ failure, or multimorbidity: A health records survey study by Willemijn Tros, Jenny T van der Steen, Janine Liefers, Reinier Akkermans, Henk Schers, Mattijs E Numans, Petra G van Peet and A. Stef Groenewoud in Palliative Medicine

sj-pdf-2-pmj-10.1177_02692163211068692 – Supplemental material for General practitioners’ evaluations of optimal timing to initiate advance care planning for patients with cancer, organ failure, or multimorbidity: A health records survey study Click here for additional data file. Supplemental material, sj-pdf-2-pmj-10.1177_02692163211068692 for General practitioners’ evaluations of optimal timing to initiate advance care planning for patients with cancer, organ failure, or multimorbidity: A health records survey study by Willemijn Tros, Jenny T van der Steen, Janine Liefers, Reinier Akkermans, Henk Schers, Mattijs E Numans, Petra G van Peet and A. Stef Groenewoud in Palliative Medicine
