# Supplementary material for: General practitioners’ evaluations of optimal timing to initiate advance care planning for patients with cancer, organ failure, or multimorbidity: A health records survey study
Source: Palliat Med. 2021 Dec 30;36(3):510–8. doi: 10.1177/02692163211068692 (PMC8972953; doi:10.1177/02692163211068692)
Supplement: sj-pdf-1-pmj-10.1177_02692163211068692 – Supplemental material for General practitioners’ evaluations of optimal timing to initiate advance care planning for patients with cancer, organ failure, or multimorbidity: A health records survey study [file sj-pdf-1-pmj-10.1177_02692163211068692.pdf]

**Supplementary file 1. the demographical questionnaire filled out by participating GPs**

GP characteristics

1. What is your gender?
    a. Male
    b. Female
    c. Other

2. What is your age?
   *Your answer has to be between 18 and 90*
   *Integer only*
   ……… years

3. How long have you been practicing as a general practitioner?
   *Your answer has to be between 0 and 90*
   *Integer only*
   ……… year(s)

4. What is your additional expertise?
   I have a specialization / additional training in the field of:
   *Multiple answers possible*
    a. Care for older people
    b. Palliative care
    c. Chronic illness (e.g. COPD, cardiovascular disease, Diabetes)
    d. No additional expertise
5. Other, i.e. …

Practice characteristics

1. What type of practice do you work at?
    a. Solo practice
    b. Solo practice within a primary care facility
    c. Duo practice
    d. Duo practice within a primary care facility
    e. Group practice
    f. Group practice within a primary care facility

2. What applies to you?
    a. A am employed (salaried service)
    b. I am self-employed (independent)
    c. Other, i.e. …

3. How is your practice situated?
    a. Rural
    b. Semirural/suburban

c. Urban

4. What is the size of the practice?
   *Your answer has to be a minimal of 0*
   *Integer only*
   The practice serves … patients
