# Supplementary material for: General practitioners’ evaluations of optimal timing to initiate advance care planning for patients with cancer, organ failure, or multimorbidity: A health records survey study
Source: Palliat Med. 2021 Dec 30;36(3):510–8. doi: 10.1177/02692163211068692 (PMC8972953; doi:10.1177/02692163211068692)
Supplement: sj-pdf-2-pmj-10.1177_02692163211068692 – Supplemental material for General practitioners’ evaluations of optimal timing to initiate advance care planning for patients with cancer, organ failure, or multimorbidity: A health records survey study [file sj-pdf-2-pmj-10.1177_02692163211068692.pdf]

Supplementary file 2. examples of the content of a patient health record displayed in the online environment

-2 jaar | -1½ jaar | -1 jaar | -½ jaar | †

Brief

Datum 21 augustus 2013

K77 - Decompensatio cordis

> Thuiszorg/hartfalenzorg/ vpk …: mv. zegt zich goed te voelen. Dyspnoe: vermindert bij vorige keer. Vermoeidheid is toegenomen.
>
> Vraagstelling: kramp/pijn in de vingers en handen. Jicht?
>
> Gesproken over NTBR?

Herhalingsrecept

Datum 25 september 2013

Medicatie

Middel Bumetanide tablet 1mg
Dosis 2D1T

Middel Bumetanide tablet 2mg
Dosis 2D1T

Herhalingsrecept

Datum 25 september 2013

Medicatie

Middel Clopidogrel tablet fo 75mg (waterstofsulfaat)
Dosis 1D1T

-2 jaar -1½ jaar -1 jaar -½ jaar

Visite huisarts langer dan 20 min

Datum 9 oktober 2013

Subjectief

K77 - Decompensatio cordis

halfjaarlijkse controle ivm DC. overdracht thuiszorg op 21/8. Gaat goed behoudens kleine actieradius, status quo, moeite met accepteren van beperkingen, ook blijft rugpijn onderrug aawezig, wisselend, vooral bij lang staan. pcm helpt dan niet.wil nog zeker 5 jr blijven leven, lijkt nog niet echt doordrongen van de ernst van haar hartfalen

Objectief

K77 - Decompensatio cordis

RR 90/60 -> geen last van duizeligheid, dikke benen wv kouse. SaO2 99%

Plan

K77 - Decompensatio cordis

gesproken over NTBR, maar wil nog geen keuze maken, weet het nog niet. 16/10 lab, op basis daarvan verder beleid, anders volgende co door mij in april 2014

Meetwaarden

Meting	Uitslag
natrium	141 (135-145)

-2 jaar -1½ jaar -1 jaar -½ jaar †

Herhalingsrecept

Datum 25 september 2013

Medicatie

Middel Isosorbidedinitraat tablet mga 20mg
Dosis 1D1T

Middel Valsartan tablet omhuld 80mg
Dosis 1D1T

Middel Metoprolol tablet mga 200mg (succinaat)
Dosis 1D1T

Middel Pantoprazol tablet msr 40mg
Dosis 2D1T

Ik denk dat deze informatie bijdraagt aan de beslissing om het ACP-moment te plannen, want:

Visite huisarts langer dan 20 min

Datum 9 oktober 2013

Subjectief

K77 - Decompensatio cordis

halfjaarlijkse controle ivm DC. overdracht thuiszorg op 21/8. Gaat goed behoudens kleine actieradius,

status quo, moeite met accepteren van beperkingen, ook blijft rugpijn onderrug aawezig, wisselend, vooral

bij lang staan. pcm helpt dan niet.wil nog zeker 5 jr blijven leven, lijkt nog niet echt doordrongen van de

ernst van haar hartfalen

-2 jaar | -1½ jaar | -1 jaar | -½ jaar | †

Ik denk dat dit het ideale ACP-moment is, want:

Kunt u in uw eigen woorden, in een paar zinnen omschrijven wat u zou bespreken in het ACP-moment?

Beoordeling voltooien

Visite huisarts langer dan 20 min

Datum 17 april 2014

Subjectief

K77 - Hartdecompensatie

halfjaarlijkse controle bezoek: gaat redelijk stabiel, lichte achteruitgang in ADL en actieradius, verleden maand slechter, toen bij pacemaker controle HF van 60 naar 70 en nu weer wat beter. Krampen in de handen, vervelend met puzzelen

Plan

K77 - Hartdecompensatie

vw vit D def start suppeltie, op proef magnesium tab tegen krampen, maalox max 2dd ivm slechte nier, voor 2 wk op proef
